# Assessing the effects of human activities on the foraging opportunities of migratory shorebirds in Austral high-latitude bays

**DOI:** 10.1371/journal.pone.0212441

**Published:** 2019-03-13

**Authors:** Juan G. Navedo, Claudio Verdugo, Ignacio A. Rodríguez-Jorquera, José M. Abad-Gómez, Cristián G. Suazo, Luis E. Castañeda, Valeria Araya, Jorge Ruiz, Jorge S. Gutiérrez

**Affiliations:** 1 Bird Ecology Lab, Instituto de Ciencias Marinas y Limnológicas, Universidad Austral de Chile, Valdivia, Chile; 2 Estación Experimental Quempillén, Chiloé, Facultad de Ciencias, Universidad Austral de Chile, Ancud, Chile; 3 Ecología y Evolución de Enfermedades Infecciosas, Instituto de Patología Animal, Universidad Austral de Chile, Valdivia, Chile; 4 Centro de Humedales Río Cruces, Universidad Austral de Chile, Valdivia, Chile; 5 Conservation Biology Research Group, Universidad de Extremadura, Badajoz, Spain; 6 Department of Animal Ecology and Systematics, Justus Liebig University Giessen, Giessen, Germany; 7 Programa de Genética Humana, Instituo de Ciencias Biomédicas, Facultad de Medicina, Universidad de Chile, Santiago, Chile; 8 Centro de Estudos do Ambiente e do Mar (CESAM), Departamento de Biologia Animal, Faculdade de Ciências da Universidade de Lisboa, Lisbon, Portugal; MARE – Marine and Environmental Sciences Centre, PORTUGAL

## Abstract

Human presence at intertidal areas could impact coastal biodiversity, including migratory waterbird species and the ecosystem services they provide. Assessing this impact is therefore essential to develop management measures compatible with migratory processes and associated biodiversity. Here, we assess the effects of human presence on the foraging opportunities of Hudsonian godwits (*Limosa haemastica*, a trans-hemispheric migratory shorebird) during their non-breeding season on Chiloé Island, southern Chile. We compared bird density and time spent foraging in two similar bays with contrasting disturbance levels: human presence (mostly seaweed harvesters accompanied by dogs) was on average 0.9±0.4 people per 10 ha in the disturbed bay, whereas it was negligible (95% days absent) in the non-disturbed bay. Although overall abundances were similar between bays, godwit density was higher in the non-disturbed bay throughout the low tide period. Both days after the start of the non-breeding season and tidal height significantly affected godwit density, with different effects in either bay. Time spent foraging was significantly higher in the non-disturbed bay (86.5±1.1%) than in the disturbed one (81.3±1.4%). As expected, godwit density significantly decreased with the number of people and accompanying dogs in the disturbed bay. Our results indicate that even a low density of people and dogs can significantly reduce the foraging opportunities of shorebirds. These constraints, coupled with additional flushing costs, may negatively affect godwits’ pre-migratory fattening. Hence, as a first step we suggest limiting human presence within bays on Chiloé to 1 person per 10 ha and banning the presence of accompanying dogs in sensitive conservation areas.

## Introduction

Globally, around 30% of coastal wetlands have been lost as a result of recent human activity [1], although this may be underestimated given the ongoing degradation of large coastal areas in ‘developing countries’ [2]. Particularly, increases in human population density near estuaries have resulted in a number of adverse effects, including infilling, the construction of dykes and drainage systems, and the conversion of land for agricultural and residential uses [3]. In addition, human exploitation of intertidal resources has become highly intensive in many coastal areas around the world [4]. Besides direct stock reductions of target species (e.g. bivalves or crustaceans; [5, 6]), exploitation may also affect non-target organisms, which are often responsible for key benthic processes [7]. Moreover, human activities can reduce the overall available area for wildlife via disturbances [8]. Hence these practices are a potential source of conflict between commercial and conservation interests [9]. However, such traditional activities are an important source of income for livelihoods, probably learned from ancestors and thus belonging to the human and natural heritage of coastal areas [10], and overall have a lower impact [11] than mechanical (modern) ones [12]. Assessing the impact of traditional activities at coastal areas is therefore essential to develop adaptive management measures that make them compatible with wetland biodiversity (from benthic invertebrates to waterbirds), and the ecosystem services they provide [13].

Migratory shorebirds are an essential component of the biodiversity *sensu lato* [14]. They are highly susceptible to disturbances [15], as they tend to inhabit wetlands that are discrete, patchily-distributed, and relatively small [16]. In this context, estuaries and bays are of crucial importance for the conservation of migratory shorebird populations throughout the world. Despite this, a recent review reported that the global conservation status of sandpipers and allies (Fams. Charadriidae and Scolopacidae) has deteriorated since the 1970s [17]. Among sandpipers’ populations with known trends 70% are decreasing, with some of them falling under the conservation status of Globally Endangered [17]. Large numbers of many shorebird species are concentrated in a few areas during the non-breeding season (review in [16]). These key areas are essential for shorebirds that need to significantly increase their body mass at the end of the non-breeding season in order to fuel non-stop northbound flights of several thousand kilometers [18]. Therefore, increasing level of human disturbances associated with non-regulated socio-economic and/or recreational activities at key sites represents an important potential threat to migratory processes [6].

It is well established that human presence can cause disturbance to shorebirds in a variety of ways, especially during the non-breeding season [19]. For instance, disturbances can reduce foraging budget, increase energetic costs, limit access to profitable areas, and promote the risk of predation of shorebirds [20, 21]. Several studies have directly assessed the responses of non-breeding shorebirds to a given source of disturbance in a field setting [11; 22–27], while others have modelled the potential effects of different disturbance scenarios [28–30]. Typically, the response of shorebirds to disturbance depends on the magnitude and frequency of the disruption. For example, shorebirds have been observed to avoid a disturbed site within a wetland [31], or even to leave it definitively in response to frequent disturbances [32]. By contrast, some shorebirds can develop habituation to a source of disturbance depending on the perceived level of risk and frequency of disturbance [33], specific habitat traits [34], and the existence of alternative functional habitats [35]. Despite the existence of both empirical and theoretical approaches to understand the effects of disturbance on shorebird populations (see also [36]), a broad limitation on this knowledge is that the numbers of animals that would use these sites in the absence of disturbance are generally unknown [37]. In addition, a further limitation is imposed by the fact that most wetlands are not pristine when studied, but already modified ones.

In Chile, harvesting of red algae (*Agarophyton chilensis;* [38], locally known as ‘pelillo’) has been carried out in intertidal areas since ancient times (see [39]) as a traditional activity to fertilize coastal crops. However, the increasing price of *A*. *chilensis* in international markets for agar production has promoted the interest for its cultivation. This is carried out in the same intertidal areas traditionally used for the extensive extraction after the collapse of natural seaweed beds during the 1980’s [40]. Locally, *A*. *chilensis* is an important economic resource for coastal inhabitants [10], involving several people (often accompanied by dogs) that work by hand and often use oxcarts to transport collected seaweed up for stocking in supratidal areas. Despite the potential disturbances associated to this traditional activity, no study has investigated its effects on wildlife.

**Fig 1:**
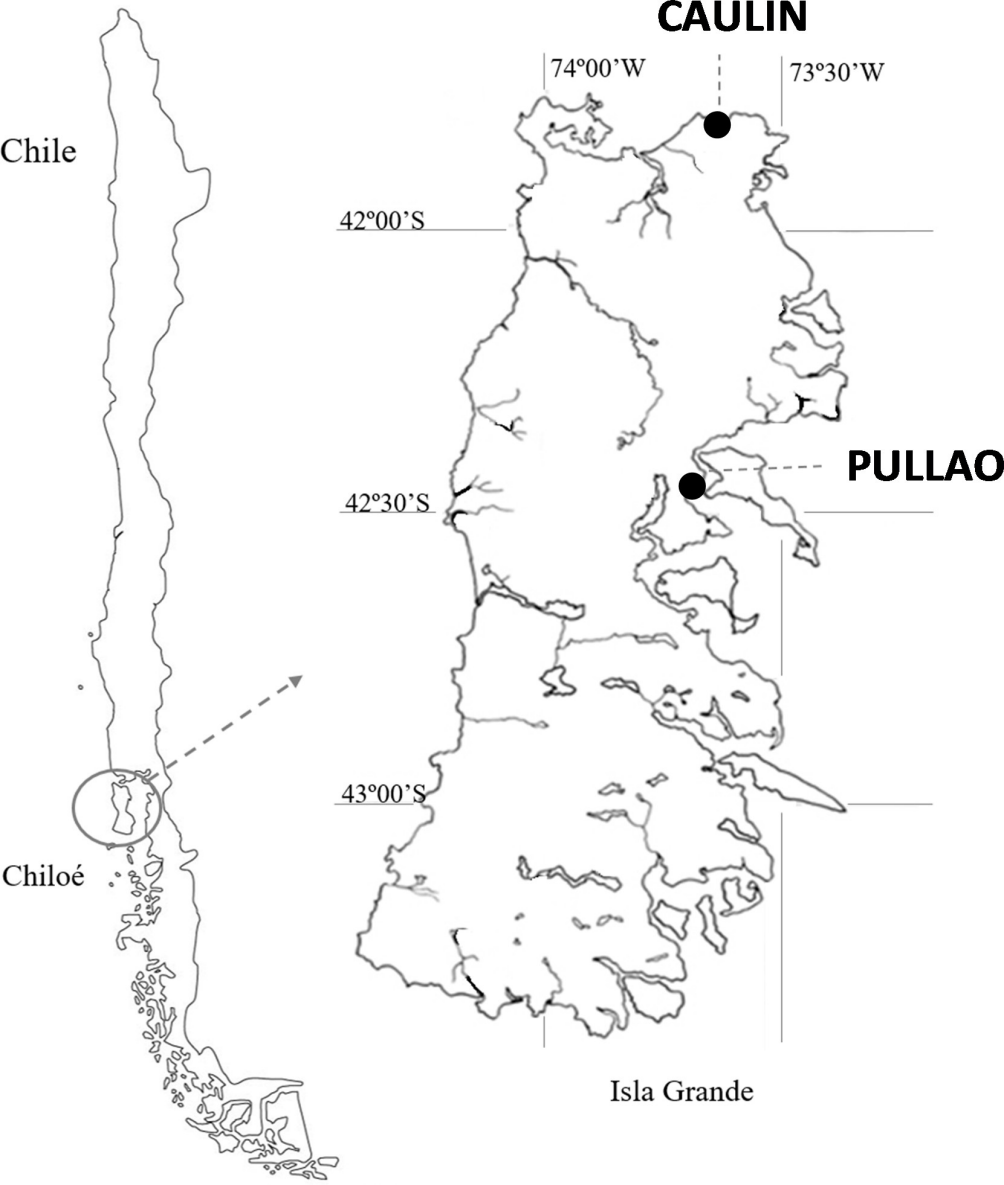
Location of the two bays selected for this study within Chiloé Island (see text for details). Caulín (disturbed bay); Pullao (non-disturbed bay).

Chiloé Island (southern Chile; Fig 1) is a key site on the East Pacific Flyway still lacking human settlements or human activities in some bays [41]. Thus, it offers an advantageous field setting to study the potential effects of human activities on shorebirds. Here, we assessed the effects of human presence, mainly associated to traditional seaweed culture, on the abundance and foraging activity of Hudsonian godwits (*Limosa haemastica*, a long-distance migratory shorebird) during their non-breeding season in soft-bottom intertidal areas with contrasting levels of human disturbance. Because the presence of potential disturbance sources during the low-tide period can reduce the available area for birds to forage, as well as increase vigilance and flushing responses to approaching stimuli [42], we predict that total foraging time and abundance of foraging birds would be significantly reduced in the disturbed area. We further predict that bird density would decrease with the number of people in the disturbed area. The objectives of the study were two-fold: First, to contribute to better understanding the indirect effects of human presence on shorebirds’ foraging activity over intertidal flats; and second, to propose management recommendations for traditional activities towards the reduction of potentially negative effects on migratory populations, which could also be applied to other coastal wetlands worldwide.

## Methods

### Study area and model species

Chiloé Island (Fig 1) occupies a strategic location on the East Pacific Flyway, providing foraging grounds for thousands of migrating shorebirds. During the Austral summer, this area supports the largest non-breeding populations of different shorebird species that breed in North America and spend the non-breeding season on the Southern Pacific coast of America. Notably, it holds *ca*. 21,000 Hudsonian godwit (hereafter godwits) and *ca*. 5,000 Whimbrel (*Numenius hudsonicus*) [43], among other migratory and resident shorebirds [44] some of global conservation concern [45]. Consequently, in 2011, an area composed by several small bays supporting an important fraction of these populations received recognition as a Hemispheric Site (highest concern) within the Western Hemisphere Shorebird Research Network [46].

To evaluate the effects of human disturbance on shorebirds’ foraging opportunities, we selected two bays with similar available intertidal area and shoreline length but contrasting human pressure. Caulín (disturbed bay) is located in the northern part of Chiloé (Fig 1), and holds an effective intertidal foraging area for shorebirds (*sensu* [47]) of 2.7 km of shoreline length and about 101 ha during spring tides. Several human activities such as seaweed culture, traditional shellfishing, and tourism take place in this bay [48]. Pullao (non-disturbed bay) is located in the central east part of Chiloé (~70 km apart; Fig 1) and is part of WHSRN Hemisphere Site. It is a shallow bay with a low level of human development and no seaweed culture but some people occasionally harvesting seaweed uplift (i.e. seaweed remains that the tides deposit at the upper intertidal limit). Pullao holds an effective intertidal area of 2.4 km of shoreline length and about 112 ha during spring tides. This bay is one of the areas that support more godwits within Chiloé Island during high tides [43]. Noticeably, a recent study showed that overall macrobenthic biomass is higher in Pullao than in Caulín [49]. However, polychaete biomass, the main food supply for godwits at Chiloé (J.G. Navedo pers. obs) and elsewhere [50, 51], is similar in both bays [49].

We selected the Hudsonian godwit as a model species because: (i) it is the most abundant shorebird in the intertidal areas of the island [43]; (ii) its foraging activity is restricted to intertidal areas during low-tide (J. Valenzuela and J.G. Navedo pers. obs.); (iii) its daily energy requirements significantly increase during the last part of the non-breeding season before embarking on a non-stop migratory flight of *ca*. 10,000 km [52]; and (iv) it is a species of high conservation concern along the Americas [53]. Along with the global importance of Chiloé, it thus represents an exceptional model to explore the potential effects of human presence derived from a widespread traditional activity on the conservation of migratory shorebird populations.

We followed Ethics Law in Chile. No specific permissions were required for these locations/activities, since sampling was made by observations from distance and field studies did not involve endangered or protected species in the country.

### Study design

The study was conducted throughout two consecutive non-breeding seasons (2013–2014 and 2014–2015), from October-November (i.e. when birds arrive on Chiloé) to February-March (i.e. when they depart back to breeding grounds in Alaska) [52]. In both bays, we divided the intertidal area into four different sectors with a width of 500 m. The limits of these sectors were perpendicularly demarcated to the shoreline using wooden sticks. Since godwits are basically tide-followers while foraging (*sensu* [54]), we used abundance as a proxy of bird density at each sector. We conducted monthly surveys at both study sites during consecutive days in order to minimize potential differences associated with tidal amplitude and other environmental factors. An observer (always the same person) arrived at the corresponding bay at least 30 minutes before the first count. The observer counted godwits, people and accompanying dogs every 30 minutes at each sector during the central part of the low-tide period (from 2 hours before to 2 hours after the low-tide peak). After each count, foraging activity was estimated. To do so, the behaviour of each bird was observed and categorized as feeding or non-feeding (for details see [11]). In the very few cases that godwits were present in a sector but there were less than 30 birds (godwits are gregarious foragers), we excluded the foraging activity in the analyses. For flocks of up to 500 birds, we randomly selected three separated subgroups and estimated the foraging activity of 30 individuals in each group. Population-level foraging activity (percentage of actively foraging birds; [11; 25; 55]) was finally determined for each count and sector during the low tide period by adding up the activity of each recorded individual (see [55]). Since tides are semi-diurnal on Chiloé [56], available foraging time during the daily diurnal low-tide period is c. 5.5–6.5 hours depending on moon phase. Using the conservative value (5.5 hours) to estimate minimum biological differences, total average time devoted to forage during daylight can be therefore estimated by multiplying available foraging time by average foraging activity. Peregrine falcons (*Falco peregrinus*) occasionally disturbed shorebirds during our observations, so observations made after falcon attacks (or when it was present in the area) were removed from the analyses.

### Statistical analyses

Godwit counts in our dataset exhibited more zeros than a Poisson or negative binomial distribution could handle. Therefore, we analyzed the abundance using a zero-inflated negative binomial (ZINB) model, which included the effect of site (disturbed vs. non-disturbed bay) and tidal period (i.e. each 30-minute count) (fixed factors) on the occurrence and abundance of godwits (response variables). To test for potential temporal effects, we included the days after 20 October (i.e. the onset of the austral summer field season; hereafter ‘daysafter’) as a covariate. Daily tidal amplitude (tidal height estimated in cm over Lower Low Water; www.shoa.cl) was also included as a covariate to control for the effect of intertidal foraging area on godwits’ foraging activity. The additive and multiplicative (interaction) effect of predictors on godwit abundance were evaluated by comparing ΔAIC scores. The model with lower AIC score was compared with candidate models by using Wald test. The activity of godwits was analyzed as a proportion of active individuals over inactive (i.e. total *minus* actives) using generalized linear models (GLM) with a quasi-binomial error distribution and logit link, which accounts for overdispersion. Interaction between fixed effects (site and tidal period) and covariates (‘daysafter’ and tidal height) were analyzed in a stepwise fashion using *F*-test.

Finally, we examined the quantitative effects of the presence of people and accompanying dogs on godwit abundance and foraging activity within the disturbed bay using a ZINB and a GLM model with a quasibinomial error distribution, respectively. Since the number of people and dogs was highly correlated (*r* = 0.78, *p* <0.001), we simply used number of people as an explanatory variable in order to avoid collinearity. All analyses were performed using the software R version 3.3.0 (R Development Core Team 2013).

## Results

Both bays were consistently used as foraging areas by a similar fraction of the godwit population, with an overall average (±SE) of 1,503.5 ± 52.7 (n = 126; range 60–2,420) and 1,506.2 ± 103.4 godwits (n = 136; range 0–4,030), respectively, at each count in Caulín and Pullao throughout the low tide period. In Caulín (disturbed bay) we recorded an average presence of 9.3 ± 3.6 people·count^-1^ (range 0–70; n = 126). Most people were dedicated to seaweed culture (72%) and shellfishing by hand (21%), with the remaining (7%) dedicated to walking or other recreational activities. Seaweed harvesters were often (30% of the counts) accompanied by dogs, with an average of 0.9 ± 0.4 dogs·count^-1^ (range 0–11) within the bay. By contrast, in Pullao (non-disturbed bay) we detected on average 0.5 ± 0.2 people·count^-1^ (range 0–4; n = 136), with people absent in 95% of counts (n = 516). With the exception of a single day, where one tourist with two dogs was observed during two counts on one edge of the bay, people were exclusively dedicated to shellfish or seaweed collection by hand (without oxcarts and accompanying dogs).

**Fig 2:**
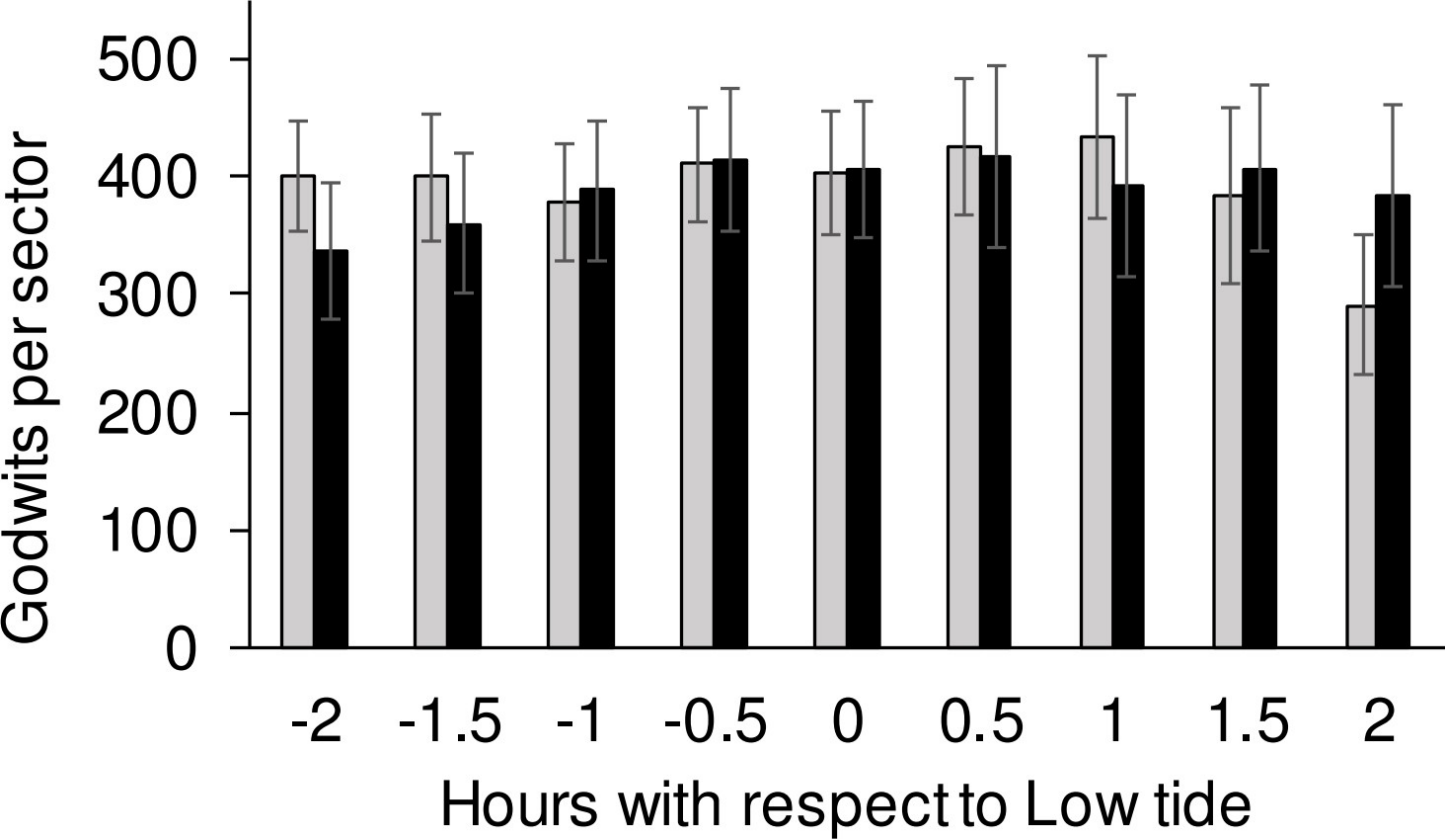
Variation (means ± SE) in Hudsonian godwit abundance within a fixed sector length (a proxy of density) throughout the low-tide period (i.e. 4 hours) in the disturbed (grey bars) and non-disturbed bay (black bars) (see text for details) over the 2-year study.

**Fig 3:**
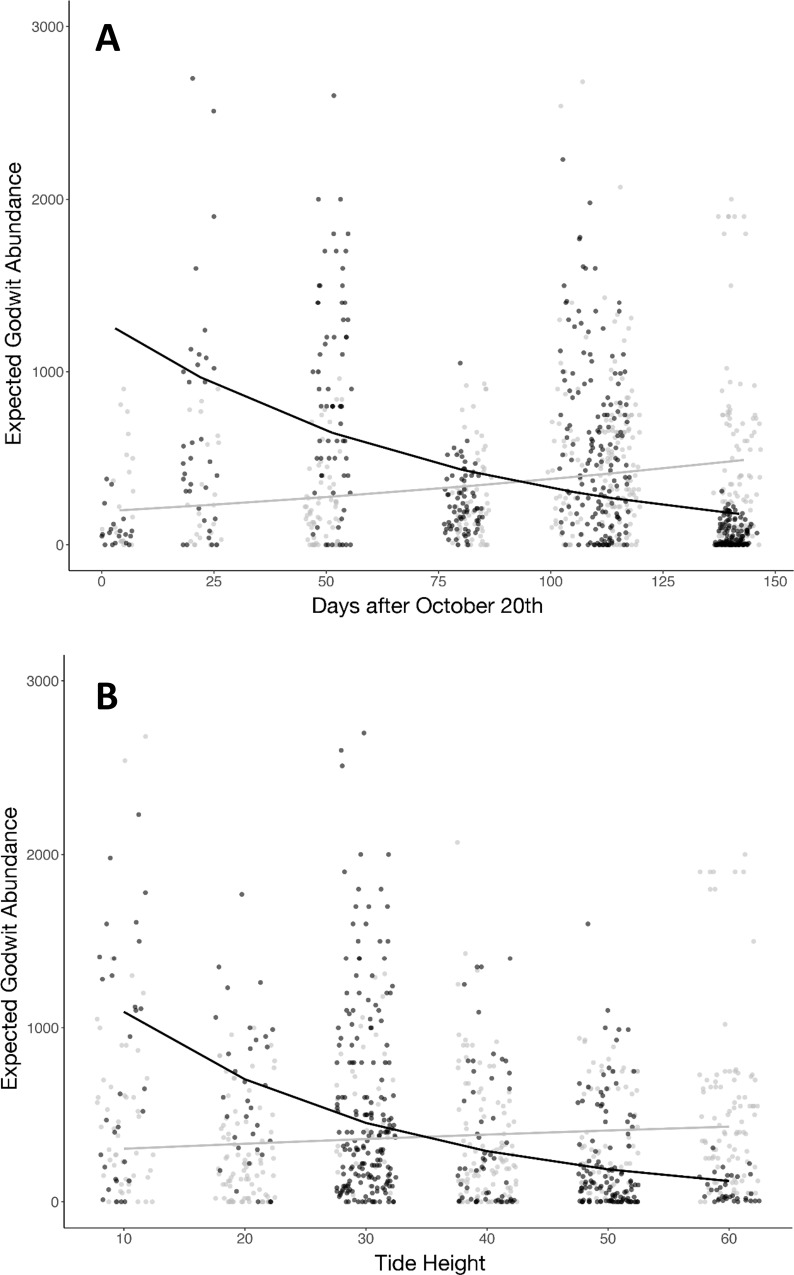
Expected godwit abundance within a fixed sector length (a proxy of density) during the low-tide period in the disturbed (grey line) and non-disturbed bay (black line) (see text for details) in relation to (A) Days after October 20^th^; (B) Tide height. Note that values close to 0 are referred to spring tides and close to 60 are referred to neap tides.

When counts at different sectors and other predictive variables were considered, results of the GLM indicated that there were significant differences in godwit density between the two sites, being consistently higher in the non-disturbed bay (Table 1). Density was significantly different throughout the low tide period at both sites, with a higher density during the peak of the low tide period with respect to both the final ebbing and initial rising of the tide, and no significant interaction between bay and tide period (Z = -1.529; *p* = 0.126; Fig 2). Both ‘daysafter’ and tidal height significantly affected godwit density, but they had a different effect between bays as indicated by their significant interactions (Table 1). Godwit density decreased throughout the season in the non-disturbed bay whereas it remained similar in the disturbed bay (Table 1; Fig 3A). However, godwit density decreased in the non-disturbed bay as tidal height decreased, and the opposite relationship was found in the disturbed bay (Table 1; Fig 3B). These interactions between bays with daysafter and height, along with tide period, were the only variables retained in the most parsimonious model explaining variation in godwit density with an equally-supported model (ΔAIC = 0.11, *X*^2^ = 1.89, *p* = 0.168) also including the interaction between bay and tide period. All remaining variables were not retained into these models.

**Table 1:** Generalized linear model showing the effects of site (disturbed and ‘non-disturbed bay’), tide period (hours with respect to low tide), ‘daysafter’ (number of days after October 20^th^) and tide height (tidal amplitude), and their interactions on Hudsonian godwit abundance in two bays within Chiloé Island (see text for details).

	Estimate	SE	Z	p
site	1.609	0.204	7.875	***
tide period	0.472	0.074	6.411	***
daysafter	0.005	0.001	3.676	***
tide height	-0.024	0.007	-3.137	**
site x daysafter	-0.017	0.002	-8.624	***
site x tide height	0.038	0.012	3.206	**

** p < 0.01

*** p < 0.001

**Fig 4:**
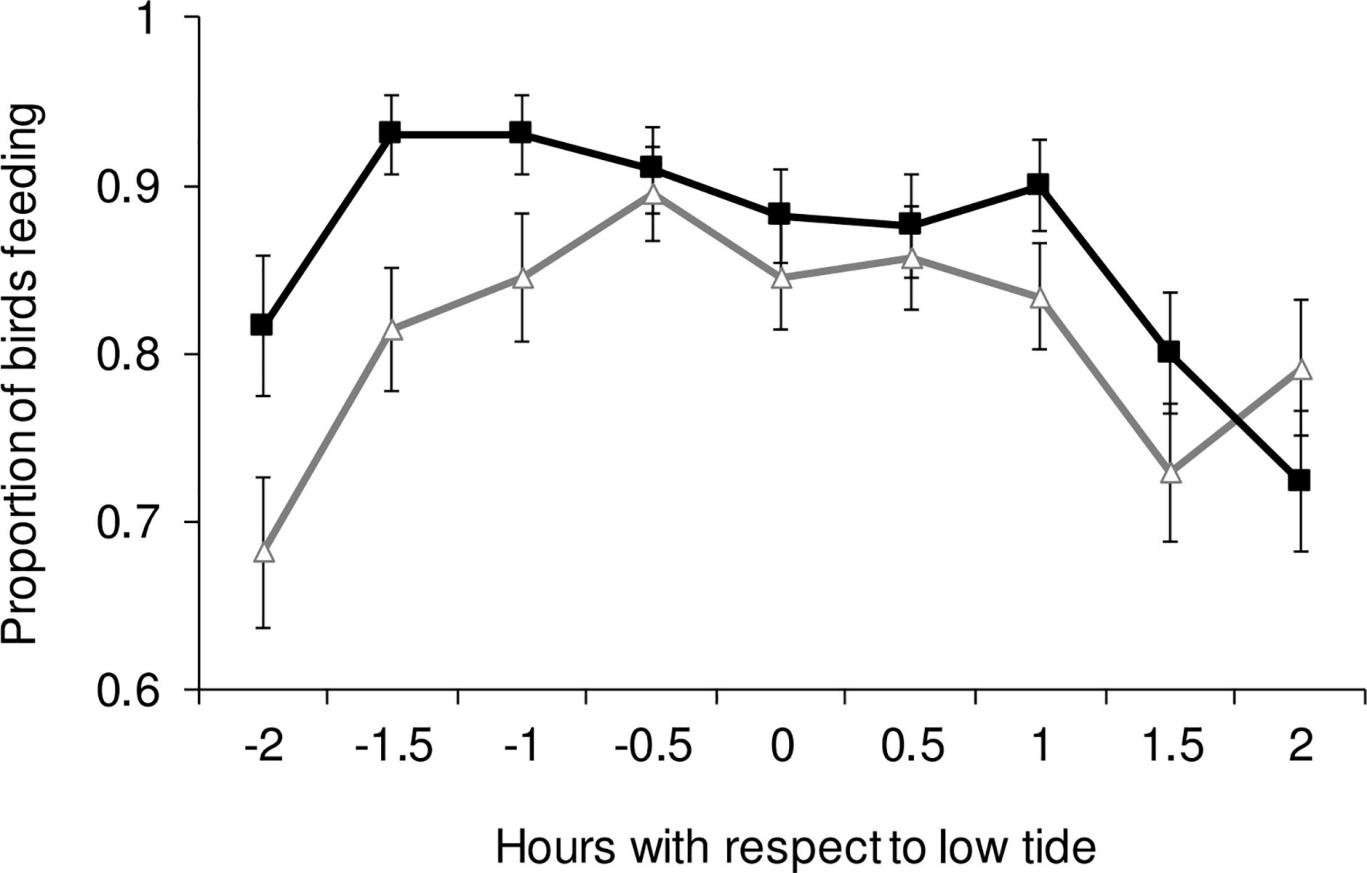
Variation (means ± SE) in Hudsonian godwit foraging activity (%) throughout the low-tide period (i.e. 4 hours) in the disturbed (open triangles and grey line) and non-disturbed bay (full squares and black line) (see text for details) over the 2-year study.

Mean foraging activity of godwits during low tide was significantly higher in the non-disturbed bay (86.5 ± 1.1%) than in the disturbed bay (81.3 ± 1.4%) (*F*_1,587_ = 10.98; *p* < 0.0001). Foraging activity was significantly different throughout the low-tide period (*F*_7,579_ = 6.59; *p* < 0.0001), being lower at the beginning and at the end of this period (Fig 4). There was no significant interaction between bay and tide period (*F*_8,578_ = 1.44; *p* = 0.17). Finally, ‘daysafter’ had a significant effect on birds’ foraging activity (*F*_8,578_ = 14.00; *p* < 0.0001), with an increase during the final period of the non-breeding season at both sites. Neither tidal height nor any interaction term showed a significant effect on godwits’ foraging activity.

**Fig 5:**
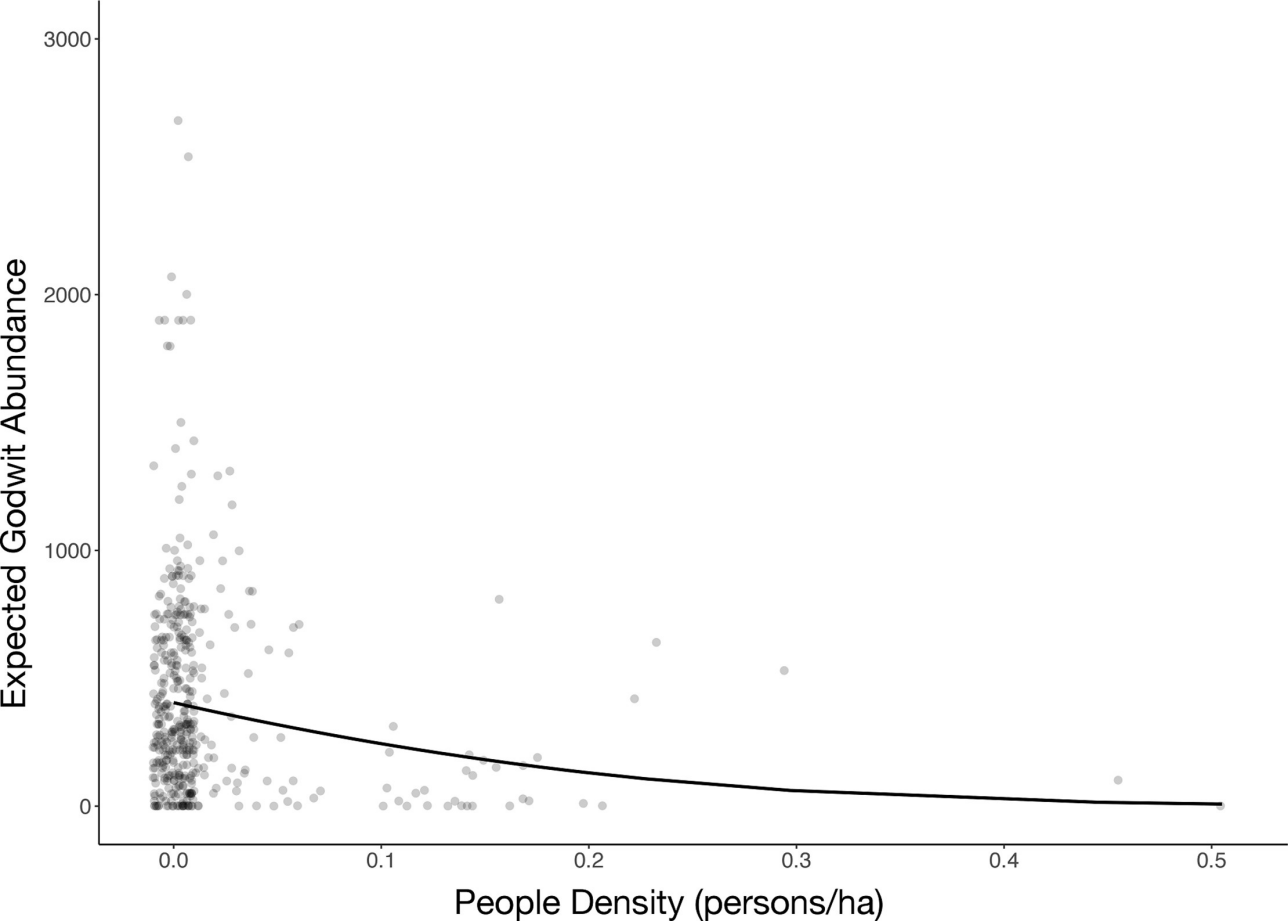
Expected abundance of Hudsonian godwits as a function of the number of people present in the disturbed bay. Note observed data (grey dots) superimposed (jitter plot), whereas the line represents the predicted values from the ZINB model (see text for details).

Godwit density significantly decreased as the number of people increased in the disturbed bay (*Z* = 4.12; *p* < 0.0001; Fig 5), but no effect was found on the foraging activity of birds (*t* = 1.391; *p* = 0.165).

## Discussion

The most remarkable finding of the present study is that a traditional activity entailing presence of people working with artisanal methods can significantly affect the distribution and foraging behavior of avian top predators in coastal ecosystems. Our results support the notion that even a relatively low human disturbance pressure is enough to significantly reduce the density and foraging activity of godwits throughout the diurnal low-tide period in a disturbed bay. More importantly, we found consistently reduced godwit density and foraging activity in the disturbed bay in comparison to an undisturbed bay. Our comparative approach accounts for one of the commonest limitations of studies testing human-driven disturbances in the field, i.e. that the number and/or behavior of animals that would use sites in the absence of disturbance is generally not known [37]. Whether the reduction in bird density and foraging activity have an effect on an animal’s fitness will likely depend on its specific vulnerability, the magnitude and duration of the disturbance source, the existence of alternative foraging areas during low tide, weather conditions, and the species’ functional response [27; 29; 57; 58].

On Chiloé, godwits foraging in a bay with a relatively intensive seaweed culture at the lower part of intertidal area lost 5% of active foraging time per diurnal low-tide period. Therefore, considering 330 min (i.e. 5.5 h) of diurnal low-tide, godwits actively forage on average during 285 min in the non-disturbed bay and during 268 min in the disturbed one, which means a 17-min loss per diurnal low-tide period. This reduction would probably have no effect on the individual fitness of other waterbirds (i.e. swans, gulls, waterfowl) that also forage during high tide (e.g. [59]). However, coastal migratory shorebirds have relatively high levels of energy expenditure [60] and have only a limited time to find their food and meet their high energy requirements [61; 62]. Since foraging time is restricted to low-tide and seaweed harvesters operate on a daily basis [i.e. except during neap tide periods), godwits would need to leave the area and move to other smaller bays nearby [see 43], or to compensate for this loss of foraging time (e.g. by feeding more efficiently) to avoid any cumulative effect, particularly during energetically demanding periods.

We recorded consistent godwit densities throughout the season in the disturbed bay. In addition, godwits did not have access to any supratidal supplementary foraging area on Chiloé (J. Valenzuela pers comm.) or could not extend total foraging time during low tide (this study). However, they might be able to compensate for a 5% loss of foraging time [29], for example by increasing overall intake rate during nocturnal foraging [63]. Indeed, during periods of increased energetic demands such as the pre-migratory fattening, many long-distance migratory shorebirds need to increase overall daily intake rate to be able to double their body mass prior to departure [64], and some species do so by increasing time devoted to nocturnal foraging [65]. Based on data from GPS-tagged individuals, godwits consistently forage at night on Chiloé throughout the season (J.G. Navedo unpubl. data). For this reason, during pre-migratory fattening already time-constrained godwits [66] could hardly compensate for any loss of foraging time during low tide period. The overall increase in foraging activity of godwits in both studied bays by the end of the non-breeding season supports this view.

Furthermore, birds experience energy and time costs associated to flight initiation [42; 67] and these costs may be important for godwits as they flush in response to an approaching disturbance, such as seaweed harvesters and especially their accompanying dogs (J. G. Navedo pers obs). As foraging time is an essential limiting factor for shorebirds to optimize intake rate [68], these additional costs can additionally reduce opportunities for godwits to reach their high daily energy requirements during pre-migratory fattening. Since body condition is significantly correlated with individual survival in long-distance migratory shorebirds [69–71], human activities can thus be affecting individual fitness of godwits using bays of Chiloé with a relevant seaweed pressure. For example, such activities might result in lower body condition at departure or migration delays towards breeding grounds, effects which can be difficult to compensate for over the course of the annual cycle [66; 72].

Besides the reduction in available foraging time in the disturbed bay, godwit density was lower compared to the non-disturbed bay. Such a reduction in overall abundance in the disturbed bay was more intense as the number of people with accompanying dogs increased. Although we did not accurately measure predation risk, a key driver of shorebird distribution (see [73]), it is likely to be similar in both areas as we observed two and three peregrine attacks in each bay throughout the systematic surveys. Regarding food supply, overall polychaete biomass (the main prey for godwits) was similar between bays [49]. Therefore, presence of people, mainly by means of current seaweed culture, is the most likely factor explaining the reduction in godwit density and foraging activity observed during low tide in the disturbed bay. This is also supported by the significant increase in godwit abundance during neap tide periods in this bay, when human presence associated to seaweed activity is significantly reduced or even absent.

### Conservation implications

A density of <0.9 people per 10 ha (i.e. 3.4 people per km of shoreline) with accompanying dogs was enough to reduce the abundance and foraging time of godwits in a disturbed bay, thus hampering overall carrying capacity of key austral non-breeding bays for shorebirds (see [74] for a Palearctic example). This might indirectly increase bird density in other bays, and some individuals with lower abilities might be forced to forage in suboptimal foraging patches (i.e. with lower food supply and/or high predation risk; [73]). Further studies are needed to assess whether godwits can offset the energy and time costs of an apparently non-invasive traditional activity, or whether these costs could promote carry-over effects in this and other species. Individual godwits can, to some extent, dissipate deviations during the annual cycle owing to an effective foraging at highly productive non-breeding grounds [52; 66]; however, scheduling of northbound migratory movements cannot be delayed [75]. Therefore, human-driven disturbances on Chiloé may entail fitness consequences in the long term for individuals using similar disturbed bays.

Despite the recently international recognition of Chiloé as Hemispheric Site within the WHSRN [46], several activities that may negatively affect shorebird populations have expanded rapidly during the last decade [41]. Yet, comprehensive studies on the impact of human activities such as aquaculture are still lacking [53]. We therefore encourage coastal managers and local stakeholders to limit the presence of seaweed (and other) harvesters within WHSRN bays of Chiloé to the capacity threshold of 1 person per 10 ha (i.e. the average pressure recorded in this study, rounded values), with a mid-term goal of reducing it and testing its effectivity as an adaptive management measure. We also recommend banning the presence of dogs accompanying either traditional harvesters or tourists, as proposed a decade ago for any sensitive conservation area [76], such as those within the WHSRN. The reasoning behind this recommendation is that dogs have evolved as top predators in many ecosystems and their presence alone induces anti-predator responses in birds, including vigilance and early flight [77], driving reductions in bird abundance of more than 40% in some areas [76]. Both measures will help to make compatible an important traditional activity with essential migratory processes which are also potential complementary sources for local economies, such as small-scale tourism initiatives.

These measures are a first step towards sustainable management of an important traditional activity in a commercially exploited wetland [40] of international importance for shorebirds. Although we have not measured effects on godwit fitness, if we are to protect migratory species and the migration phenomenon proactive conservation measures (e.g. [78]) are needed while populations are still abundant [79]. These may aid the conservation of the tribe Numeniini (Fam. Scolopacidae), where godwits belong, with seven out of 13 species Near Threatened or Globally Threatened, including two Critically Endangered [80].
